# Quantitative comparison of 2D and 3D late gadolinium enhancement MR imaging for cardiomyopathies

**DOI:** 10.1186/1532-429X-16-S1-P330

**Published:** 2014-01-16

**Authors:** Fabian Morsbach, Sonja Gordic, Robert Götti, Markus Niemann, Hatem Alkadhi, Gruner Christiane, Robert Manka

**Affiliations:** 1 Institute for Diagnostic and Interventional Radiology, University Hospital Zurich, Zurich, Switzerland; 2 Cardiology Clinic, University Hospital, Zurich, Switzerland

## Background

LGE is widely used as a means to quantify scar or fibrotic tissue in patients suffering from cardiomyopathies. In clinical routine 2D data acquisition is most commonly practiced, albeit having the drawback of multiple breath-holds and long acquisition times. 3D acquisition can significantly reduce acquisition time. This leads to shortened scan time and a more efficient use of available MRI resources. So our purpose was to determine whether the quantification of myocardial fibrosis in patients with Fabry disease and hypertrophic cardiomyopathy (HCM) using a late gadolinium enhancement (LGE) single-breath-hold three-dimensional (3D) inversion recovery magnetic resonance (MR) imaging sequence is comparable with a clinically established two-dimensional (2D) multi-breath-hold sequence.

## Methods

40 consecutive patients (18 men; mean age 50 ± 17) with either Fabry disease (n = 18) or HCM (n = 22) were enrolled in this prospective study. Studies were conducted on a 1.5-T clinical MR imaging system. Spatial resolution was the same for 3D and 2D images (field-of-view, 350 × 350 mm2; in-plane-resolution, 1.2 × 1.2 mm2; section-thickness, 8 mm). Datasets were analyzed for subjective image and quantitative evaluation of myocardial mass (grams), fibrotic mass (grams) and total fibrotic tissues percentage. Statistical analysis included Wilcoxon-signed-rank test, student's t-test for paired samples and Bland-Altman analysis.

## Results

There was no significant difference in subjective image quality between acquisitions (P > 0.1) for either disease. In patients with Fabry disease there was no significant differences in myocardial mass between 3D (100.7 g ± 30.8 g) and 2D acquisition(99.9 g ± 31.9 g; P = 0.55), as well as for fibrous tissue mass(3.9 g ± 6.4 g vs 4.0 ± 6.4 g; P = 0.89) and total fibrous percentage(3.4% ± 5.5%vs3.4 ± 5.5;P = 0.89). Bland-Altman analysis showed good agreement between 3D and 2D datasets for myocardial mass(mean difference: 0.8 g; limits of agreement: -10.2 g - 11.8 g), fibrous tissue mass (mean difference: -0.02 g; limits of agreement: -1.45 g-1.41 g), total fibrous percentage (mean difference:0.02%; limits of agreement: -1.31%-1.35%). In patients with HCM there was no significant differences in myocardial mass between 3D (115.5 g ± 33.3 g) and 2D acquisition (116.7 g ± 33.6 g;P = 0.48), as well as for fibrous tissue mass (5.6 g ± 8.6 g vs 5.7 g ± 8.7 g;P = 0.6) and total fibrous percentage (4.3% ± 6.4% vs 4.3% ± 6.5%;P = 0.89). Bland-Altman analysis showed good agreement between 3D and 2D datasets for myocardial mass (mean difference: -1.2 g;limits of agreement:-16.1 g - 13.7 g), fibrous tissue mass(mean difference -0.08 g;limits of agreement: -1.33 g - 1.17 g), total fibrous percentage(mean difference:-0.01 g;limits of agreement:-1.01 g-0.99 g). Acquisition time was significantly shorter for 3D sequences (24.9 seconds ± 5.2 seconds) as compared to 2D sequence (349.1 seconds ± 62.3 seconds, P < 0.001).

## Conclusions

3D LGE imaging enables comparable quantification of fibrous myocardial tissue compared to a 2D sequence at a faster acquisition rate.

## Funding

Nothing to disclose.

